# Why biology must prioritize data reanalysis in the era of artificial intelligence

**DOI:** 10.1371/journal.pbio.3003888

**Published:** 2026-07-22

**Authors:** Daniel Ghaderi, Ali Sahimi, Benjamin Esser, Kelly V. Ruggles, Roy Maimon

**Affiliations:** 1 Department of Medicine, New York University, New York City, New York, United States of America; 2 Department of Biomedical Engineering, New York University, Tandon School of Engineering, New York City, New York, United States of America

## Abstract

New technologies have generated an unprecedented volume of biological data, much of which remains underutilized. This Perspective argues that the next era of biological discovery is likely to emerge from AI-driven data reanalysis, but realizing its full potential will require standards, validation, and responsible use.

## Teaser

New technologies have generated huge volumes of biological data, much of which is underutilized. AI-assisted data reanalysis could drive the next era of biological discovery, but realizing its full potential requires standards, validation, and responsible use.

Scientific revolutions do not only emerge from new experiments, but often from new ways of interpreting existing observations. For example, Johannes Kepler transformed astronomy through rigorous reanalysis of observational data collected by Tycho Brahe, revealing the laws of planetary motion before their underlying mechanisms were understood.

Over the past decade, single-cell transcriptomics, spatial omics, and large-scale sequencing technologies have redefined what is technically possible to measure, generating biological datasets of unprecedented scale and complexity [1,2]. Concurrently, public repositories, open-access resources, and FAIR data practices have enabled the wide-scale sharing, integration, and reuse of biological datasets [3,4]. This emerging data ecosystem is creating the opportunity to revisit existing datasets with new hypotheses, analytical tools, and conceptual frameworks. Yet, as biological datasets become increasingly large, complex, and accessible, our capacity to interpret them has not scaled proportionally. Published datasets are frequently underutilized after primary analysis, as investigators shift toward new data synthesis to address specific questions. Additionally, poor quality standards and incomplete metadata often discourage investigators from engaging with shared datasets. This underutilization is exemplified by a methodological survey of more than 454,200 public omics datasets, which found that only 12,162 (2.7%) had undergone at least one documented reanalysis [5]. Consequently, vast amounts of high-quality biological data accumulate in the public domain with substantial unrealized potential for discovery.

A structural shift toward data reanalysis is needed. Reanalysis involves repurposing published datasets to address new questions, reevaluate prior conclusions, and uncover biological insights beyond the scope of the original study. It increases the scientific value of existing data while reducing the need for redundant data generation. By revisiting datasets with improved computational tools [6], refined biological questions, and alternative conceptual frameworks, researchers can uncover patterns and relationships that were invisible at the time of primary analysis. Notably, significant advances in tumor biology have been made possible by robust data sharing and reanalysis paradigms. For example, integration of multi-omic data across the Cancer Genome Atlas and the Broad Institute’s Firehose platform has enabled new tumor subtype classification with implications for patient prognosis and provided molecular phenotyping of histologic tumor subtypes [7,8]. Therefore, just as Kepler extracted hidden celestial order from preexisting astronomical observations, systematic reanalysis of biological datasets may reveal overlooked cellular states, latent genetic programs, and previously unrecognized principles of human biology. In this framework, reanalysis is not secondary science, but an engine for conceptual discovery itself. From a more practical standpoint, reanalysis is less expensive than experimentation and can be conducted from virtually anywhere, lowering financial or geographic barriers to entry while also being environmentally advantageous by reducing the need for redundant, large-scale experiments. Rather than competing with new data generation, reanalysis and experimentation should be viewed as complementary activities, with each informing and strengthening the other.

But biological data today are far richer and more complex than in the era of Kepler, introducing both unprecedented scientific opportunity and substantial analytical challenges. Conventional computational approaches remain limited in their ability to extract subtle biological structure from the high-dimensional, multimodal datasets that now define modern biology. For example, cell type annotation in transcriptomic datasets remains highly sensitive to preprocessing strategies, quality-control thresholds, dimensionality reduction methods, clustering granularity, and marker selection [2]. These technical choices can profoundly influence the identification of rare or quiescent cell populations, including stem cells and immune cell subtypes, limiting reproducibility across studies. Such challenges extend across biological data modalities and increasingly constrain our ability to fully interpret large-scale datasets.

Fortunately, the very scale and complexity of high-throughput data make it inherently compatible with machine learning and artificial intelligence (AI) tools, which draw statistical power from size, variability, and cross-cohort integration. AI can be used to model nonlinear relationships, integrate heterogeneous modalities, and extract structure from high-dimensional biological space, all of which are necessary to disentangle meaning from big data. For instance, AI systems have already demonstrated the ability to reproducibly classify cells based on validated reference data, improving the reliability of annotation pipelines [9]. Beyond improving upon current analytical methods, AI opens new horizons of nuanced data interpretation such as reconstructing differentiation trajectories or modeling intercellular communication dynamics within complex microenvironments [10]. Ultimately, AI has the potential to work synergistically with reanalysis in a restructured data lifecycle that maximizes scientific insight [11]. Models can be used to identify rare, quiescent, or subtle biology that escape primary analyses, allowing for the reannotation of datasets to improve training data for subsequent models. This iterative cycle creates a progressively more accurate representation of biological systems. Importantly, AI does not replace biological reasoning; it amplifies it by enabling deeper interrogation of data already in hand.

But indiscriminate use of AI could substantially distort the research landscape. When trained on poorly powered, homogeneous, or biased datasets, model predictions can diverge from the underlying biological reality, generating inaccurate conclusions that may become amplified through iterative training cycles. In this way, small errors have the potential for pervasive impact. Furthermore, AI can be exploited to rapidly generate poor-quality analyses that further corrupt the scientific literature. A striking example emerged from the National Health and Nutrition Examination Survey, a large public health dataset that was misused to produce hundreds of formulaic analyses based on selectively sampled variables in a process suggestive of large-scale data dredging [12].

To address these risks, rigorous standards for AI-assisted data reanalysis must be established. This begins with careful selection of training datasets containing well-defined ground truth, perturbational structure, longitudinal information, and consistent metadata. Robust training data should be well-annotated, interoperable across modalities, and aligned with FAIR data principles [3]. Importantly, experimentation and primary data generation remain essential, and researchers must remain cognizant of the long-term potential for their datasets to become integrated into future AI systems. Particular attention must be paid to minimizing technical, biological, and demographic biases that may otherwise become amplified through AI-driven analyses. Additionally, data sharing must be practiced with high standards for data and metadata quality to enable reanalysis and improve algorithmic training. Simply increasing the volume of observational data does not automatically improve inference.

Furthermore, validation and reproducibility standards must ensure that machine learning models undergo rigorous testing across diverse datasets and that algorithmic outputs are not mistaken for biological truth. Contrary to concerns about human replacement, critical human reasoning becomes more important than ever in the era of AI. Scientists and clinicians must remain responsible for evaluating model reliability, interpreting biological significance, and experimentally or clinically validating computational predictions. Even in the presence of advanced AI systems, scientific judgment remains indispensable, and algorithmic outputs must ultimately be subject to human scrutiny and validation.

Finally, ethical standards must be upheld to prevent irresponsible or fraudulent use of AI that undermines scientific integrity. Because AI systems can generate misleading, biased, or low-quality findings at scale, human judgment is vital for maintaining rigor, transparency, and trust in science [13]. Accordingly, AI should not be ignored in educational settings. Instead, the next generation of biologists must be trained to use AI critically, responsibly, and with appropriate scientific skepticism.

A new era of biological discovery driven by AI-assisted data reanalysis is on the horizon. Scientific progress has historically advanced through multiple complementary paths, including experimental discovery, conceptual reinterpretation of existing observations, and the communication of transformative ideas. While modern biology continues to emphasize data generation, the coming years may require renewed focus on the equally powerful role of reanalysis. Like Kepler’s reinterpretation of astronomical observations, AI-driven reanalysis may fundamentally reshape biological discovery, but realizing its potential will require rigor, responsibility, and continued human oversight.

## Abbreviation:

AI: artificial intelligence
